# Predictive Value of the Transthoracic Echocardiography Index for Acute Kidney Injury after Cardiac Valve Surgery

**DOI:** 10.3390/jcdd9100316

**Published:** 2022-09-21

**Authors:** Juan Guo, Yugang Hu, Sheng Cao, Chuangli Feng, Xin Huang, Qing Zhou

**Affiliations:** Department of Ultrasound Imaging, Renmin Hospital of Wuhan University, Wuhan 430061, China

**Keywords:** transthoracic echocardiography index, cardiac valve surgery, acute kidney injury, nomogram, risk assessment

## Abstract

**Background:** We aimed to demonstrate whether the preoperative transthoracic echocardiography index (TTEI) could improve the predictive value of clinical parameters for cardiac valve surgery-associated acute kidney injury (CVS−AKI). **Methods:** A total of 213 patients who underwent surgical CVS at Renmin Hospital of Wuhan University were consecutively recruited in this retrospective study. TTE assessments were performed within 7 days before surgery and logistic regression was used to determine TTEI. A nomogram was constructed by integrating TTEI and clinical features, and the net reclassification index (NRI) and integrated discrimination improvement (IDI) were applied to evaluate the improvement in TTEI for CVS−AKI. **Results:** Among them, 66 patients (30.9%) developed CVS−AKI. The TTEI was calculated as follows: −6.579 + 0.068 × pulmonary artery systolic pressure (mmHg) −0.742 × LVEF (>55%, yes or no) + 0.346 × left ventricle posterior wall thickness (mm). The nomogram based on the TEEI and other clinical factors possessed excellent performance (C-index = 0.880), had great calibration and discrimination, and was clinically useful. Furthermore, NRI (0.07, 95% confidence interval, 95%CI, 0.01–0.12, *p* = 0.02) and IDI (0.08, 95%CI, 0.01–0.20, *p* = 0.02) indicated that TTEI could significantly improve the predictive value of clinical features for CVS−AKI. **Conclusions:** As a simple access and cost-effective parameter, the preoperative TTEI may be a reliable and useful factor for CVS−AKI.

## 1. Introduction

Cardiac surgery-associated acute kidney injury (CSA-AKI), which is also classified as the type 1 form of cardiorenal syndrome, is associated with an increased risk of morbidity and mortality after cardiac surgery, as well as prolonged hospital stays and higher healthcare costs [1,2,3]. Compared with other types of cardiac surgery, for example, coronary artery bypass grafting, valve surgery patients might have an increased risk of AKI and mortality partly owing to its higher cardiopulmonary bypass (CPB) and cross-clamp times [4,5]. However, little literature could be obtained about the biomarkers or predictive models, particularly for cardiac valve surgery-associated AKI (CVS−AKI) instead of just integrated as CS [6,7].

Echocardiography, which mainly consists of transthoracic echocardiography (TTE) and transesophageal echocardiography (TEE), has been widely applied in preoperative, intraoperative, and postoperative CS patients and, to some extent, TEE is currently the gold standard for diagnosis after CS [8,9,10]. Compared with TEE, TTE is more frequently used by clinicians for hemodynamic management and guides decision-making at the ‘point-of-care’ because it is non-invasive, easy, and quickly feasible [11,12,13]. Furthermore, several studies had also demonstrated that some parameters of TTE could serve as an independent predictor for AKI in patients with different types of diseases, as well as CS patients [14,15,16]. However, to the best of our knowledge, no study was conducted to investigate the association between integrated TTE parameters and CVS−AKI. Hence, in the present study, we firstly created a new TTE index (TTEI) from real-world data in our hospital that underwent surgical CVS. Furthermore, we combined the new indicators with other common clinical variables to develop sequence diagrams for predicting CVS−AKI in patients following surgical CVS.

## 2. Materials and Methods

### 2.1. Patients and Study Design

**Figure 1:**
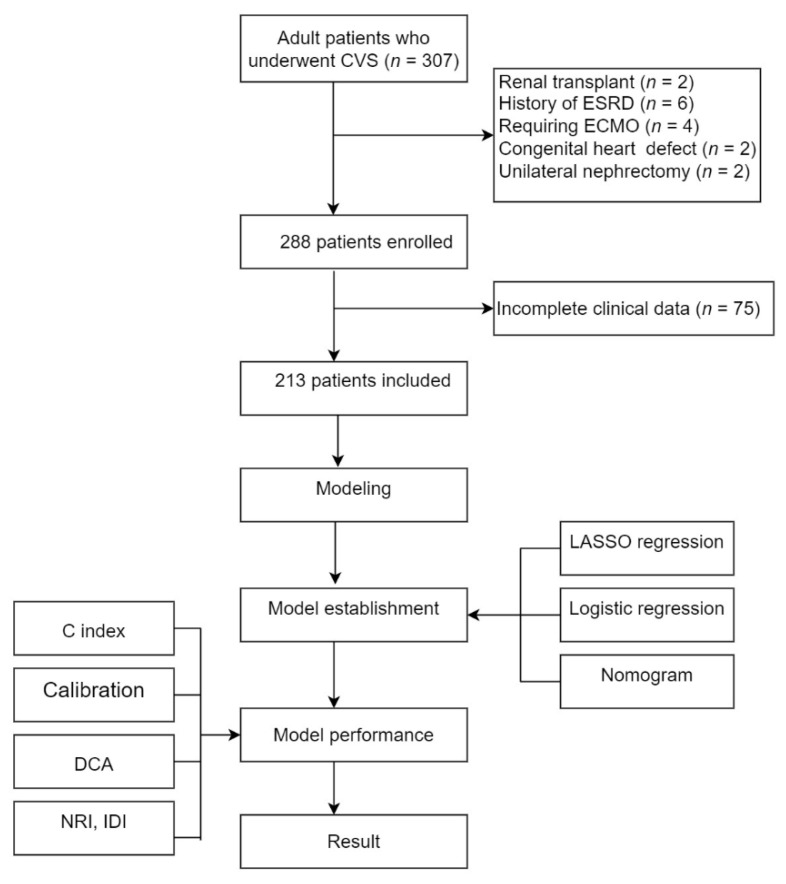
The flow chart of this study.

This is a single−center retrospective study involving 307 adult patients who underwent CVS at the Renmin Hospital of Wuhan University from 1 January 2020 to 31 December 2021. The following participants were eligible for the study: (1) adult patients who underwent surgical CVS and (2) patients who performed at least one TTE measurement within 7 days before CVS. We further excluded patients who had a history of renal transplantation (*n* = 2), unilateral nephrectomy (*n* = 2) or congenital heart defect (*n* = 4), pre-existing end-stage renal disease (*n* = 6), and patients who require extracorporeal membrane oxygenation after CVS (*n* = 5). Furthermore, we also eliminated patients with incomplete clinical data (variables with >20% missing values). Finally, a total of 213 patients were enrolled in this study (Figure 1). This study was approved by the Ethics Committee of Renmin Hospital of Wuhan University without the need for informed consent from the participants.

Demographic, clinical, and biochemical data were obtained from hospital medical records on the first day of their hospital admission.

The primary event in this study was the incidence of CVS−AKI according to the KDIGO recommendation based on serum creatinine criteria [17], which means that serum creatinine increased by 0.3 mg/dL (26.5 µmol/L) within 48 h after surgical CVS or a serum creatinine increase of at least 1.5 times the baseline level within 7 days after surgery. The estimated glomerular filtration rate (eGFR) was calculated with the initial serum creatinine measurement before surgical CVS and was estimated by the CKD-EPI equation [18].

### 2.2. Echocardiographic Parameters

TTE examinations were conducted 7 days before surgical CVS and were performed by experienced echocardiographers in the left lateral position using a Philips EPIQ 7c with a 3.2 MHz transducer (Philips Medical Systems, Andover, MA, USA).

The images and measurements were obtained according to the recommendations of both the American Society of Echocardiography and the European Association of Echocardiography [19]. Standard left ventricular (LV) diameters, septum thickness (IVST), anterior–posterior measurements of the left atrium (LASDD), and the left ventricular posterior wall (LVPW) were measured in long-axis parasternal view according to the current ASE/EACVI recommendations [20]. Left ventricular ejection fraction (LVEF) was quantified by the modified biplane Simpson’s method [21].

**Figure 2:**
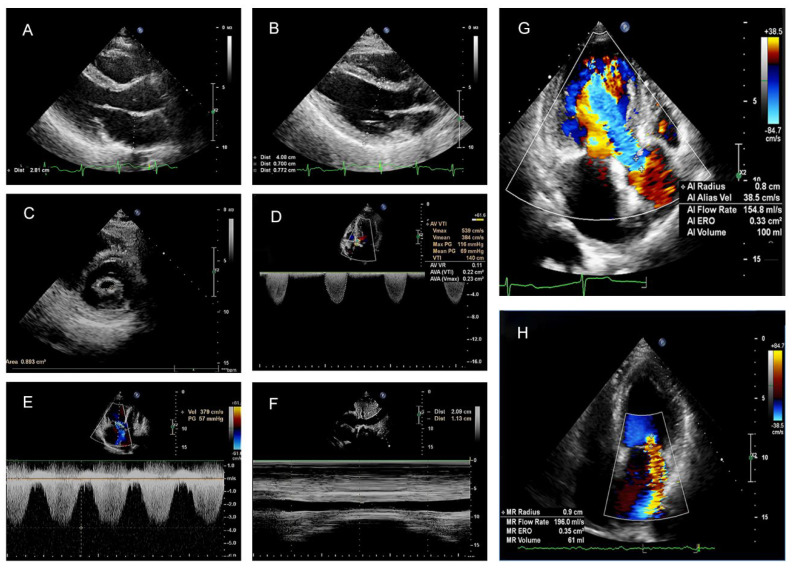
**Representation of measured values based on echocardiographic views.** (**A**) The measurements of the left atrial in parasternal left ventricular long axis (PLAX) view. (**B**) The measurements of the interventricular septum (IVS), left ventricular posterior wall (LVPW), and left ventricular end-diastolic diameter (LVEDD) in PLAX view. (**C**) Measurement of the mitral valve area in a patient with rheumatic heart disease; the mitral valve area was manually outlined and the mitral valve area is 0.89 cm^2^. (**D**) The aortic valve area was calculated using continuity equations, and the effective valvar orifice area (EOA) of the aortic valve is 0.23 cm^2^. (**E**) A continuous wave Doppler was used to measuring the end-expiration peak velocity of the tricuspid regurgitant (TR) jet. (**F**) The IVC size was measured in M-mode at the subcostal window. The IVC—CI (inferior vena cava collapsibility index) was calculated as follows: IVC-CI = [(IVC diameter in expiration—ICV diameter in inspiration)/ (IVC diameter in expiration)] × 100. Right atrial pressure (RAP) was estimated from the IVC-CI. (**G**) Quantitative assessment of aortic regurgitation using the proximal isovelosity surface area method (PISA). The effective regurgitant orifice area (EROA) is 0.33 cm^2^ and the regurgitant volume (RV) is 100 mL. (**H**) Quantitative assessment of mitral regurgitation by PISA. The EROA is 0.25 cm^2^ and the RV is 61 mL.

Pulmonary artery systolic pressure (PASP) was estimated according to the 2015 ERS/ESC Guidelines [22]. The calculation of PASP was based on the peak tricuspid regurgitant jet velocity and estimated right atrial pressure following the formula: PASP = right atrial pressure (RAP) + ΔP max, where ΔP max was calculated using the modified Bernoulli’s equation: ΔP max = 4υ², where υ is the measured peak velocity of the tricuspid regurgitation jet by continuous-wave Doppler from an apical four-chamber view. RAP was estimated by measuring the inferior vena cava (IVC) size and change with respiration. In the present study, no patients have right ventricular outflow tract obstruction, but most patients have detectable regurgitation through their tricuspid valve during systole. As per ESC guidelines, evaluation of valve regurgitation and severity of stenosis was conducted on the mitral valves, tricuspid valves, and aortic valves. Figure 2A–H shows the representation of the measured values based on echocardiographic views.

### 2.3. Statistical Analysis

All analyses were performed using R software (Version 3.6.1) and Empower (R) (www.empowerstats.com, accessed on 21 July 2021, X & Y solutions, inc. Boston MA, USA). The receiver operator characteristic curve (ROC) was used to determine the optimal cutoff value for all factors for CVS−AKI. The novel TTEI was calculated based on the β coefficients from the multivariable logistic analysis and then divided into two groups according to the optimal cutoff value. The least absolute shrinkage and selection operator (LASSO) regression was then applied to determine the significant differential factors associated with CVS−AKI. Then, univariate and multivariable logistic regression analysis was used to build a predicting model by introducing the feature selected by LASSO regression. A nomogram incorporating the important factors related to CVS−AKI was constructed with the R software. The predictive performance of the predictive nomogram for CVS−AKI was evaluated using C-index, Brier score, and calibration plots. Decision curve analysis (DCA) was used to the clinical benefits of the nomogram for CVS−AKI. Moreover, the net reclassification index (NRI) and integrated discrimination improvement (IDI) were applied to evaluate whether the TTEI could improve the predictive value of clinical features for CVS−AKI. A value of *p* < 0.05 was considered significant.

## 3. Results

### 3.1. Patient Characteristics

A total of 213 patients were finally enrolled in this study and the mean age was 58.9 years (ranging from 18−77). Of those, 66 patients (30.9%) have AKI after surgical CVS, and patients with AKI were older and had higher levels of CPB time, uric acid, and N-terminal pro-brain natriuretic peptide (NT−proBNP), PASP, IVST, and LVPW and lower levels of eGFR and LVEF than patients without AKI. AKI was more frequent in patients with hypertension and diabetes mellitus (Table 1).

### 3.2. Transthoracic Doppler Echocardiography Index (TTEI)

**Figure 3:**
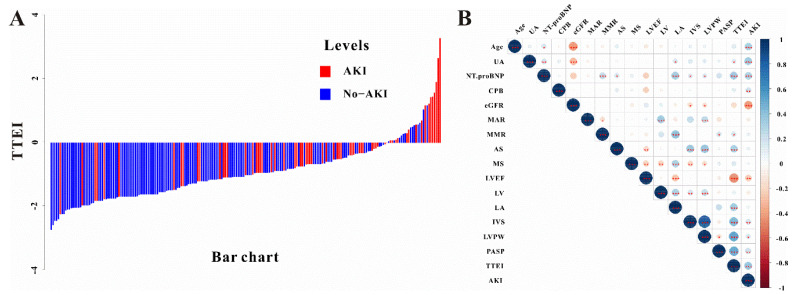
(**A**) The waterfall plot of TTEI value for each patient and (**B**) the relationship between TTEI and other TTE parameters and clinical features. * *p* < 0.05, ** *p* < 0.01, *** *p* <0.001.

TTEI was calculated based on the ten parameters obtained from TTE by multivariable logistic regression. As shown in Table 2, LVPW, LVEF > 55%, and PASP were found to be independent risk factors for CVS−AKI. Therefore, based on the β coefficients, the TTEI was calculated as follows: −6.579 + 0.068 × PASP (mmHg) −0.742 × LVEF (>55%, yes or no) + 0.346 × LVPW (mm). The TTEI for each patient presented as a waterfall plot demonstrated significant differences between AKI and no-AKI (*p* < 0.001, Figure 3A). As described in Figure 3B, the level of TTEI was significantly correlated with some parameters of TTE as well as clinical factors.

**Figure 4:**
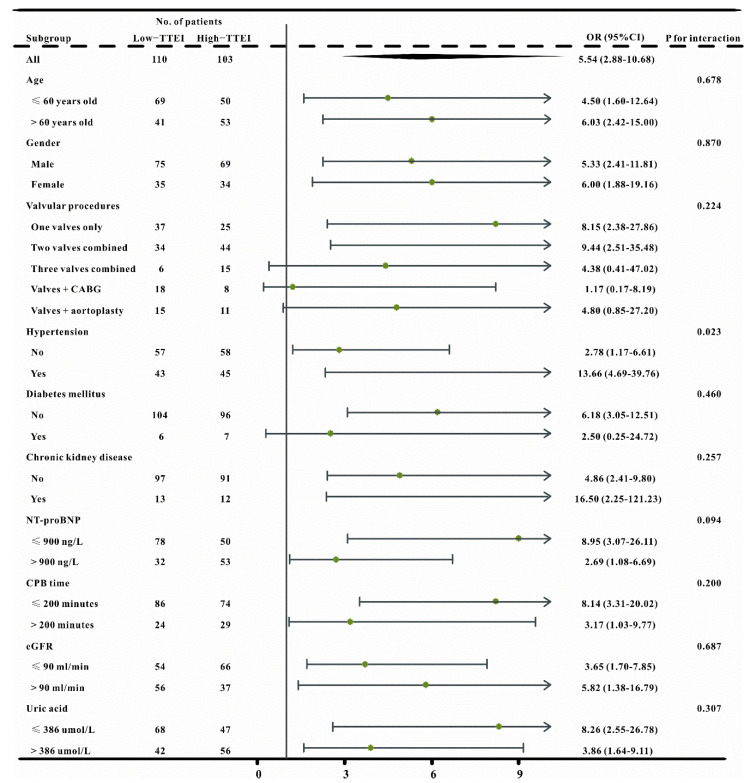
The forest plot revealed the results of subgroup analysis for CVS−AKI based on low and high TTEI groups in the crude cohort.

Based on the Youden index, all patients were divided into two groups according to the optimal cutoff value (−1.01). Patients in the high TTEI group had a higher risk of CVS−AKI (OR, 5.54, 95%CI, 2.88–10.68, *p* < 0.001) and the crude OR was 2.71 (95%CI 1.83–4.01, *p* < 0.001) when the TTEI value was used as continuous variables. The TTEI also showed a favorable predictive efficacy, with an AUC of 0.734 (95%CI 0.669–0.792; sensitivity, 75.8%; specificity, 64.0%). Moreover, in the pre-specified subgroup analysis, patients with TTEI ≤ −1.01 had a lower risk of CVS−AKI than those with TTEI > −1.01 group in almost all subgroups (Figure 4).

### 3.3. Model Construction Based on TTEI Established by LASSO Regression and Clinical Features

**Figure 5:**
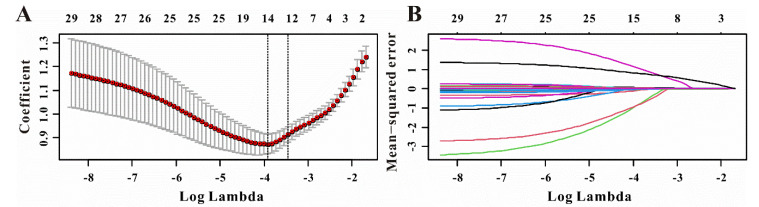
Selection of significant factors associated with CVS−AKI by LASSO logistic regression model. (**A**) Identification of tuning parameter (λ) in the LASSO model. (**B**) Profiles of LASSO coefficient for clinical features.

**Figure 6:**
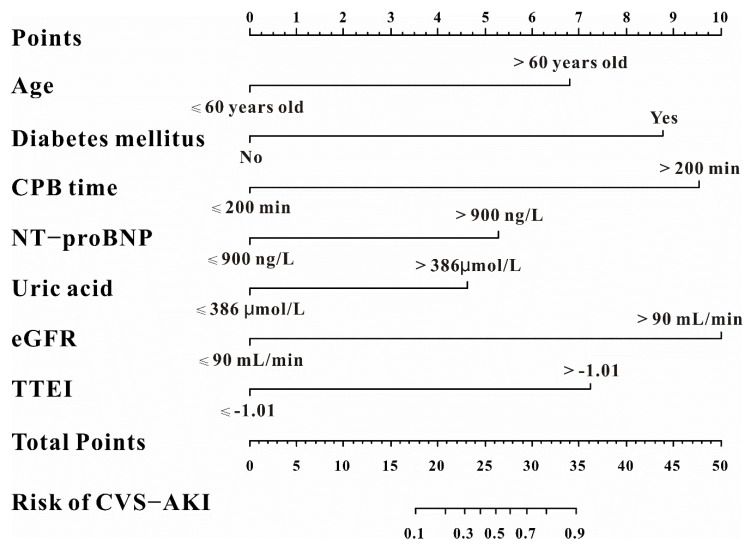
The predictive nomogram for CVS−AKI.

Of the 29 factors including TTEI and clinical features, 12 potential predictors with nonzero coefficients in the LASSO regression were selected in this study (Figure 5A,B). To provide a nomogram for CVS−AKI to clinicians that is relatively easy-to-use and highly accurate, univariate and multivariable logistic regression analysis was then used to build a predicting model by introducing the features selected in the LASSO regression. As exhibited in Table 3, age, diabetes mellitus, CPB time, NT-proBNP, uric acid, eGFR, and TTEI finally showed a significant association with CVS−AKI. Therefore, introducing the above seven independent factors, a CVS−AKI risk nomogram was developed and presented in Figure 6. Furthermore, Supplementary Figure S1 shows the violin figures of the above features in the AKI and no-AKI groups.

### 3.4. The Performance of the Nomogram for CVS−AKI

**Figure 7:**
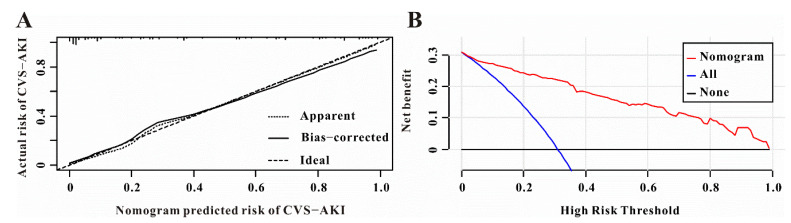
Calibration and clinical utility of the predictive nomogram. (**A**) The predictive nomogram exhibited a high correlation between the actual probability and predicted probability for CVS−AKI. (**B**) Decision curve analysis for the predictive nomogram to predict CVS−AKI in this study.

For the predictive model, the C-index of the nomogram was 0.880 and the Brier score was 0.101, which indicated a relatively great performance (Table 4). The calibration curve of the nomogram to predict the CVS−AKI also showed good agreement (Figure 7A). Hence, the nomogram of the model had a good prediction ability. The DCA showed that it would be more accurate to use this nomogram to predict the risk of CVS−AKI when the risk threshold probability was almost more than 0% (Figure 7B), which indicated that the nomogram was clinically useful.

### 3.5. The Improvement of TTEI for CVS−AKI

To determine whether TTEI could materially improve the predictive value of clinical features for CVS−AKI, NRI and IDI were used in this study. As presented in Table 4, the addition of TTEI could significantly improve the risk reclassification (NRI value, 0.07, 95%CI 0.01–0.12, *p* = 0.020; IDI value, 0.08, 95%CI, 0.01–0.20, *p* = 0.023) of CVS−AKI compared with the clinical features alone.

## 4. Discussion

In this study, we established the preoperative TTEI for the first time to predict the postoperative CVS−AKI from real-world data of 213 patients and found that TTEI could significantly improve the predictive value of clinical features for CVS−AKI. The cutoff value of −1.01 was a good threshold for the risk of CVS−AKI in almost all subgroups. We further developed a predictive nomogram integrating TTEI and other clinical factors with great calibration, discrimination, and clinically usefulness. Furthermore, the risk reclassification of the TTEI value, as measured by the NRI and IDI, was also significantly improved through the addition of clinical features. Taken together, those results suggest that TTEI was a reliable marker for CVS−AKI, and the proposed nomogram based on TTEI resulted in an accurate prediction for the recognition of patients with CSA-AKI.

CS might be the second-most common pathogenesis of AKI (following sepsis) and the morbidity rate ranges from 5% to 42% in general medicine. Michael et al. conducted a retrospective study of 801 patients with pre-existing chronic kidney disease who underwent transcatheter aortic valve replacement (TAVR) or surgical aortic valve replacement (SAVR), and found that the incidence of AKI was 11.9% (47/399) and 38.4% (154/402) in patients with TAVR and SAVR, respectively, based on the RIFLE-AKI stage system [23]. Similarly, using the data from the MIMIC-IV database, Li et al. performed a retrospective study of 7181 critical care patients with CS and concluded that the incidence of CSA-AKI was 72.0% based on the KDIGO-AKI criteria [24]. Compared with the aforementioned studies, the overall incidence of CVS−AKI in the present study was 30.9%, which was in accordance with previous studies, and the difference in the morbidity in different studies may at least partly be explained by the severity of the disease and differences in patient populations, AKI definitions, and types of surgery.

Although the American College of Radiology Appropriateness Criteria gives ultrasound the highest rating in the initial evaluation of AKI [25], the role of ultrasound in the diagnosis and management of AKI has still been explored in limited studies, and a majority of them were about the application of the contrast-enhanced ultrasound because of the advantage that it can provide quantitative assessments of renal perfusion, which may be concerned with AKI associated with vascular or hemodynamic changes [26,27,28,29]. A recent study also investigated the association between hepatic venous and right-heart ultrasound parameters and the risk of CSA-AKI, and found that the hepatic vein flow ratios were independently associated with the development of postoperative CSA-AKI [30]. In addition to the diagnosis of AKI, the role of ultrasound in the management of AKI patients may be controversial to some extent. Several kinds of literature indicated that point-of-care ultrasound could help clinicians monitor AKI patients and predict the prognosis of patients following AKI [31,32,33]. However, Allen et al. conducted a study of 90 postoperative CS patients from a single hospital and drew the conclusion that the renal US provides little benefit in managing postoperative cardiac patients [34]. Unfortunately, considering that this was a retrospective study and the information on a renal ultrasound could not be obtained by all patients, we could not explore the value of renal ultrasound for CVS−AKI.

Cardiac and renal disease share several common bidirectional pathways, which is also named cardiorenal syndrome, and TTE or TEE is an important, cost-effective, and even routine examination before CS. More importantly, considering that TTE is an easily accessible and rapid diagnostic method, patients may get a great benefit when TTE has good predictive value for the complications after CS. Thus, in the past few decades, researchers have focused their attention on the diagnostic and prognostic role of TTE and its parameters for CS-AKI. Among them, LVEF may be the first and most accepted one for AKI. In a recent study of 3314 noncardiac surgery patients, Wang et al. confirmed that preoperative LVEF (OR, 0.91, 95%CI 0.90–0.93, *p* < 0.001) was independently associated with AKI and could improve the predictive ability of logistic regression models based on conventional clinical risk factors [35]. Another study also verified that the utilization of LVEF value could be helpful for the early identification of acute myocardial infarction patients at high risk of AKI [36]. Our study adds to evidence that preoperative LVEF may be a reliable indicator for AKI following CVS. Moreover, Yockelson et al. conducted an observational cohort study of 199 CS patients who performed TTE within 48 h following chest closure and concluded that the right ventricular systolic performance by 2D speckle-tracking echocardiography was significantly associated with AKI [37]. Despite the diagnostic importance of AKI, echocardiography also had a prognostic impact on AKI patients [38]. In our previous study, we explored the association between the usage of TTE within 24 h after the diagnosis of AKI and clinical outcomes for critical care patients and demonstrated that TTE utilization was associated with decreased risk-adjusted 28-day mortality (OR, 0.80, 95%CI 0.72–0.88, *p* < 0.001) [39]. Similar to this, Han et al. conducted a cohort study of 1300 CABG patients with a 72.0 ± 28.8 month follow-up and revealed that E/e’ in preoperative echocardiography was the best predictor for long-term all-cause mortality (hazard ratio, 1.9, 95%CI 1.3–2.8) [40]. However, because of the types of cardiac diseases, about half of the patients in this study could not obtain E/e’ measurements, thus we did not include this variable in the potential predictors for CVS−AKI.

Some limitations exist in the present study. First of all, it was a retrospective, single-center study with a limited sample size. Secondly, as a retrospective study, we only included the preoperative measurements, thus intraoperative and postoperative factors, including the TTE parameters, which could influence the development of CVS−AKI, may not be included in this study because of the missing values. Thirdly, we could not obtain the measurements of renal ultrasound, thus the predictive value of the resistive index for CVS could not be explored in this study. Moreover, the diagnosis of CVS−AKI was based only on an increased serum creatinine level, instead of urinary criteria, for the reason that an indwelling urinary catheter was not placed in most of the patients enrolled in this study. Finally, we included our study population in the test cohort and validated our nomogram using bootstrap resampling, instead of splitting the dataset into two parts for development and validation. This was because, according to the TRIPOD guideline [41], the performance of the predictive model should be evaluated in some other participant dataset than the one used in its development. Finally, because of the limited sample size, we built and verified the nomogram for CVS−AKI in the same cohort, thus more prospective multi-center studies are still proposed to further verify our findings externally.

## 5. Conclusions

In the present study: We firstly revealed that the TTEI, which is a simple access and cost-effective parameter, might operate as a reliable marker for CVS−AKI. Moreover, the predictive nomogram generated by integrating the TTEI and other beneficial clinical features could potentially be a convenient and effective method for identifying patients at high risk of AKI following surgical CVS.

## Figures and Tables

**Table 1:** Comparisons of characteristics between the no AKI group and AKI group.

Characteristics	No AKI (*n* = 147)	AKI (*n* = 66)	*p*-Value
Age, years old	57.2 ± 9.3	62.9 ± 8.9	<0.001
Gender, male, *n* (%)	100 (68.0)	44 (66.7)	0.844
Types of valvular surgery, *n* (%)			0.840
One valve	43 (29.3)	19 (28.8)	
Two valves	54 (36.7)	24 (36.4)	
Three valves	13 (8.8)	8 (12.1)	
Valves + CABG	20 (13.6)	6 (9.1)	
Valves + aortoplasty	17 (11.6)	9 (13.6)	
Cardiopulmonary bypass time, minutes	162.0 ± 39.2	177.7 ± 46.8	0.012
Comorbidities, *n* (%)			
Hypertension	51 (34.7)	37 (56.1)	0.003
Diabetes mellitus	5 (3.4)	7 (10.6)	0.035
Chronic kidney disease	14 (9.5)	11 (16.7)	0.134
COPD	4 (2.7)	4 (6.1)	0.236
History of PCI	6 (4.1)	1 (1.5)	0.334
Preoperative drug usage, *n* (%)			
ACEI/ARB	38 (26.0)	19 (29.2)	0.629
NSAID	17 (11.6)	8 (12.1)	0.907
Proton pump inhibitor	81 (55.1)	39 (59.1)	0.587
Antibiotics	10 (6.8)	7 (10.6)	0.344
Usage of contrast medium recently, *n* (%)	101 (68.7)	48 (72.7)	0.556
Transthoracic echocardiography parameters			
Left ventricular end-diastolic dimension, mm	53.1 ± 8.6	51.8 ± 10.1	0.349
≤44	22 (15.0)	17 (25.8)	
>44	125 (85.0)	17 (25.8)	
Left atrial end-systolic diameter, mm	46.8 ± 12.0	47.1 ± 8.6	0.870
≤40	45 (30.6)	10 (15.2)	
>40	102 (69.4)	56 (84.8)	
Interventricular septal thickness, mm	10.6 ± 1.7	11.3 ± 1.8	0.015
≤10.5	80 (54.4)	22 (33.3)	
>10.5	67 (45.6)	44 (66.7)	
Left ventricular posterior wall thickness, mm	10.2 ± 1.5	10.8 ± 1.5	0.011
≤11.5	125 (85.0)	44 (66.7)	
>11.5	22 (15.0)	22 (33.3)	
Pulmonary arterial systolic pressure, mmHg	34.4 ± 7.4	40.7 ± 14.0	<0.001
≤43	129 (87.8)	45 (68.2)	
>43	18 (12.2)	21 (31.8)	
Left ventricular ejection fraction, %	55.5 ± 5.4	52.0 ± 6.9	<0.001
≤55	57 (38.8)	38 (57.6)	
>55	90 (61.2)	28 (42.4)	
Moderate or severe aortic regurgitation, *n* (%)	56 (38.1)	19 (28.8)	0.190
Moderate or severe mitral regurgitation, *n* (%)	62 (42.2)	34 (51.5)	0.207
Moderate or severe aortic stenosis, *n* (%)	14 (9.5)	10 (15.2)	0.232
Moderate or severe mitral stenosis, *n* (%)	15 (10.2)	9 (13.6)	0.466
Laboratory results			
White blood cells, × 10^9^/L	6.8 ± 2.3	6.6 ± 2.9	0.648
Hemoglobin, g/L	127.4 ± 21.0	128.0 ± 21.0	0.838
Platelets, × 10^9^/L	180.6 ± 68.4	188.2 ± 72.9	0.463
Albumin, g/L	39.5 ± 4.2	40.2 ± 5.1	0.283
Total bilirubin, µmol/L	16.4 ± 5.1	17.7 ± 8.9	0.455
ALT/AST	0.9 ± 0.4	1.0 ± 0.4	0.071
Blood urea nitrogen, µmol/L	7.2 ± 2.9	7.3 ± 2.6	0.745
eGFR, ml/min	91.7 ± 21.6	73.7 ± 18.0	<0.001
Uric acid, µmol/L	361.5 ± 130.9	459.5 ± 146.2	<0.001
NT-pro BNP, ng/L	361.0 (124.0, 1079.0)	2080.0 (995.0, 2993.5)	<0.001
cTnI, ng/mL	0.02 (0.01, 0.06)	0.01 (0.01, 0.03)	0.178
Potassium, mmol/L	4.0 ± 0.5	4.0 ± 0.4	0.330
Sodium, mmol/L	139.2 ± 10.1	140.7 ± 3.6	0.243
Magnesium, mmol/L	0.8 ± 0.1	0.8 ± 0.1	0.513

AKI, acute kidney injury; CABG, coronary artery bypass grafting; COPD, chronic obstructive pulmonary disease; PCI, percutaneous transluminal coronary intervention; ACEI/ARB, angiotensin-converting enzyme inhibitors/angiotensin receptor blocker; NSAID, non-steroidal anti-inflammatory drugs; ALT/AST, alanine transaminase/ aspartate aminotransferase; eGFR, estimated glomerular filtration rate; NT-pro BNP, N-terminal pro-brain natriuretic peptide; cTnI, cardiac troponin I.

**Table 2:** Logistic regression models of transthoracic echocardiography parameters for CVS−AKI.

	Univariate			Multivariate		
	β	OR (95%CI)	*p*	β	OR (95%CI)	*p*
LVEDD	−0.017	0.98 (0.95–1.02)	0.323			
LAESD	0.002	1.00 (0.98–1.03)	0.869			
IVSD	0.208	1.23 (1.04–1.46)	0.016	0.062	1.06 (0.78–1.45)	0.697
LVPW	0.257	1.29 (1.06–1.58)	0.013	0.346	1.41 (1.14–1.76)	0.002
PASP	0.058	1.06 (1.03–1.09)	<0.001	0.068	1.07 (1.04–1.11)	<0.001
LVEF > 55%	−0.762	0.47 (0.26–0.84)	0.011	−0.742	0.48 (0.25–0.90)	0.021
MAR or SAR	−0.420	0.66 (0.35–1.23)	0.190			
MMR or SMR	0.376	1.46 (0.81–2.61)	0.206			
MAS or SAS	0.529	1.70 (0.71–4.05)	0.234			
MMS or SMS	0.329	1.39 (0.58–3.36)	0.465			
Constant				−6.579		<0.001

CVS−AKI, cardiac valve surgery acute kidney injury; OR, odds ratio; 95%CI, 95% confidence interval; LVEDD, left ventricular end-diastolic dimension; LAESD, left atrial end-systolic diameter; IVSD, interventricular septal thickness; LVPW, left ventricular posterior wall thickness; PASP, pulmonary arterial systolic pressure; LVEF, left ventricular ejection fraction; MAR, moderate aortic regurgitation; SAR, severe aortic regurgitation; MMR, moderate mitral regurgitation; SMR, severe mitral regurgitation; MAS, moderate aortic stenosis; SAS, severe aortic stenosis; MMS, moderate mitral stenosis; SMS, severe mitral stenosis.

**Table 3:** Logistic regression analysis for the predictors of CVS−AKI selected by LASSO regression.

	Univariate Analysis		Multivariate Analysis	
	OR (95%CI)	*p*-Value	OR (95%CI)	*p*-Value
Age				
≤60 years old	Ref.	-	Ref.	-
>60 years old	4.29 (2.30–7.98)	<0.001	5.60 (2.23–14.05)	<0.001
Diabetes mellitus				
Yes	3.92 (1.23–12.48)	0.021	9.60 (1.39–66.37)	0.022
No	Ref.	-	Ref.	-
Chronic kidney disease				
Yes	1.90 (0.81–4.45)	0.139		
No	Ref.	-		
CPB time			11.90 (4.11–34.43)	<0.001
≤200 min	Ref.	-	Ref.	-
>200 min	3.60 (1.88–6.89)	<0.001		
History of PCI				
Yes	0.36 (0.04–3.07)	0.351		
No	Ref.	-		
Platelets				
≤171 × 10^9^/L	Ref.	-		
>171 × 10^9^/L	1.63 (0.91–2.94)	0.102		
Albumin				
≤39 g/L	Ref.	-		
>39 g/L	1.51 (0.83–2.74)	0.179		
Blood urea nitrogen				
≤6 µmol/L	Ref.	-	Ref.	-
>6 µmol/L	2.15 (1.18–3.90)	0.012	1.39 (0.57–3.38)	0.464
eGFR				
≤90 mL/min	Ref.	-	Ref.	-
>90 mL/min	0.14 (0.07–0.29)	<0.001	0.08 (0.03–0.23)	<0.001
Uric acid				
≤386 µmol/L	Ref.	-	Ref.	-
>386 µmol/L	4.20 (2.25–7.85)	<0.001	3.12 (1.30–7.48)	0.011
NT-proBNP				
≤900 ng/L	Ref.	-	Ref.	-
>900 ng/L	4.23 (2.29–7.83)	<0.001	3.84 (1.59–9.25)	<0.001
TTEI				
≤−1.01	Ref.	-	Ref.	-
>−1.01	5.54 (2.88–10.68)	<0.001	6.57 (2.50–17.23)	<0.001

CVS−AKI, cardiac valve surgery acute kidney injury; OR, odds ratio; 95%CI, 95% confidence interval; CPB, cardiopulmonary bypass time; PCI, percutaneous transluminal coronary intervention; eGFR, estimated glomerular filtration rate; NT-proBNP, N-terminal pro-brain natriuretic peptide; TTEI, transthoracic echocardiography index.

**Table 4:** NRI and IDI analyses for risk reclassification of CVS−AKI.

Outcome	AUC				IDI		NRI ^a^	
	Biomarker	Biomaker + Clinical Model	Clinical Model ^b^	*p*-Value ^c^	Value (95%CI)	*p*-Value	Value (95%CI)	*p*-Value
TTEI	0.734	0.810	0.693	<0.001	0.164 (0.108–0.219)	<0.001	0.421 (0.223–0.618)	<0.001
Nomogram excluded for TTEI	0.854	0.917		<0.001	0.231 (0.137–0.324)	<0.001	0.367 (0.182–0.552)	<0.001
Nomogram	0.880	0.925		<0.001	0.067 (0.011–0.124)	0.020	0.076 (0.014–0.196)	0.023

CVS−AKI, cardiac valve surgery acute kidney injury; AUC, area under the receiver-operating characteristic curve; IDI, integrated discrimination improvement; NRI, net reclassification index; TTEI, transthoracic echocardiography index. ^a^ The NRI is calculated through a two-way category using the event rate of CVS−AKI. ^b^ The clinical model for predicting CVS−AKI is composed of variables in Table 1 excluded from the variables in the nomogram. ^c^ Biomarker + clinical model versus clinical model.

## Data Availability

The datasets used in this study are available from the corresponding author.

## Supplementary Materials

The following supporting information can be downloaded at https://www.mdpi.com/article/10.3390/jcdd9100316/s1, Figure S1: The distribution of selected factors by LASSO regression in AKI and No-AKI group.
