# Supplementary figures and images for: Predictive Value of the Transthoracic Echocardiography Index for Acute Kidney Injury after Cardiac Valve Surgery
Source: J Cardiovasc Dev Dis. 2022 Sep 21;9(10):316. doi: 10.3390/jcdd9100316 (PMC9604519; doi:10.3390/jcdd9100316)

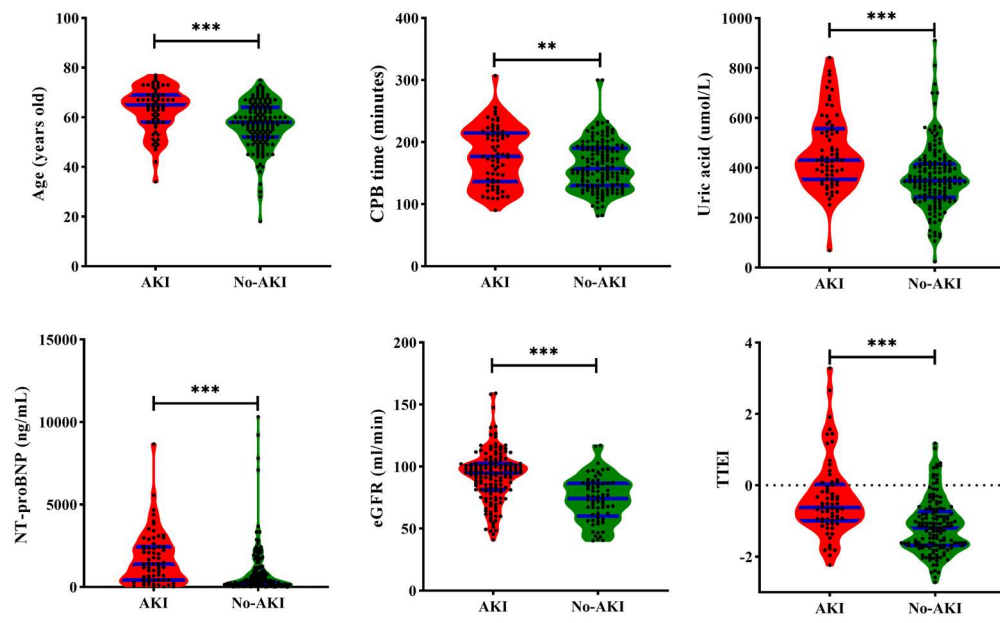

**Figure S1.** The distribution of selected factors by LASSO regression in AKI and No-AKI group.

Supplement: Supplementary file 1 [file jcdd-09-00316-s001.zip › jcdd-1893674-supplementary.pdf]
